# Results of Operating the 2025 “Heat-related Illness Emergency Department Surveillance System”

**DOI:** 10.56786/PHWR.2026.19.42.2

**Published:** 2026-11-05

**Authors:** Jisuk Chae, Eunji Jung, Daeshik An, Younjhin Ahn

**Affiliations:** Division of Climate Change and Health Hazard, Department of Health Hazard Response, Korea Disease Control and Prevention Agency

**Keywords:** Extreme heat, Heat stress disorders, Heat stroke, Public health surveillance, Climate change

## Abstract

**Objectives:**

This study aimed to analyze the characteristics and health impacts of heat-related illnesses during the summer of 2025 in the Republic of Korea and to provide evidence for prevention and response measures to minimize heat-related health risks associated with climate change.

**Methods:**

Since 2011, the Korea Disease Control and Prevention Agency (KDCA) has operated the nationwide “Heat-related Illness Emergency Department Surveillance System.” Between May 15 and September 25, 2025, data on patients with heat-related illnesses and suspected heat-related deaths were collected from 517 participating emergency departments through the “Disease and Health Integrated Management System.” These data were analyzed based on major epidemiological characteristics, including sex, age, type of illness, region, and place of occurrence. Daily surveillance results were publicly released on the KDCA website.

**Results:**

A total of 4,460 heat-related illness cases and 29 suspected deaths were reported in 2025. Compared with 2024, the cases increased by 20.4%, whereas suspected deaths decreased by 14.7%. Most patients were male (79.7%), with individuals aged 50–59 years accounting for the largest proportion of cases (19.4%). Older adults aged ≥65 years accounted for 30.1% of all cases and 58.6% of deaths. Heat exhaustion was the most common diagnosis (62.0%), and most incidents occurred outdoors (79.2%), particularly in workplaces (32.1%) and farms (12.2%) or fields.

**Conclusions:**

Heat-related illnesses predominantly affected older adults and outdoor workers during the summer of 2025. Strengthening prevention measures targeting vulnerable populations, timely dissemination of public information, and interagency collaboration are needed to reduce the growing health impacts of heat waves associated with climate change.

**Key messages**

**① What is known previously?**

Climate change continues to increase heatwave risks, and the Korea Disease Control and Prevention Agency has operated a nationwide heat-related illness surveillance system since 2011.

**② What new information is presented?**

During the summer of 2025, 4,460 heat-related illness cases were reported, representing a 20.4% increase compared with the previous year, along with 29 suspected deaths. Cases occurred predominantly among older adults and outdoor workers.

**③ What are implications?**

Heat-related health impacts were concentrated among older adults and outdoor workers, highlighting the need to strengthen prevention and response measures.

## Introduction

According to the World Meteorological Organization (WMO), 2025 was 1.44℃±0.13℃ warmer than the pre-industrial period (1850–1900), ranking among the top three warmest years on record. Furthermore, the past 11 years (2015–2025) were all recorded as among the warmest years since observations began in 1850, and in particular, the recent three years (2023–2025) ranked 1st to 3rd among the warmest years of all time, with the unprecedented global warming trend continuing [1,2].

In 2025, early heat began in the Republic of Korea in late June, and the nationwide average temperature during the summer season (June–August) was 25.7℃, which was 0.1℃ higher than that of 2024 (25.6℃), the hottest year on record, breaking the highest record since observations began in 1973. This temperature was 2.0℃ higher compared with the climatological normal temperature [2].

According to a 2025 joint report by the World Health Organization (WHO) and the WMO, as the frequency and intensity of heatwaves increase due to climate change, workers repeatedly exposed to hazardous high-temperature environments, particularly manual laborers in sectors such as agriculture, construction, and fisheries, have been shown to already be experiencing health impacts, such as heat stroke, dehydration, renal dysfunction, and neurological disorders. In addition, the report stated that serious health problems are also occurring among vulnerable groups (children, the elderly, and low-income groups) in developing countries [3]. Health damage caused by heatwaves is not limited to developing countries and is being widely reported in regions such as Europe and Japan. In 32 European countries, approximately 62,775 heat-related deaths occurred in the summer of 2024 [4]. In Japan, the number of emergency transports due to heat illness from May to September 2025 reached 100,510, recording the highest level since the start of the survey in 2008 [5].

The Korea Disease Control and Prevention Agency (KDCA) has been operating the “Heat-related Illness Emergency Department Surveillance System” since 2011 to minimize health damage to the public caused by heatwaves. In addition, by disclosing prompt occurrence information daily and key characteristics on the KDCA website through monitoring of heat-related illness occurrences, it raises public awareness and encourages adherence to prevention guidelines. This report aims to present the major operational results and implications of the 2025 summer “Heat-related Illness Emergency Department Surveillance System.”

## Methods

Since 2011, the KDCA has operated the “Heat-related Illness Emergency Department Surveillance System” to promptly identify the occurrence trends of heat-related illnesses caused by heatwaves and key characteristics, including vulnerable groups, and compiles and publishes this information on its website daily at 16:00 by region and key occurrence characteristics (by sex, age, occupation, disease, and time and place of occurrence).

In 2025, among the medical institutions nationwide that operate an emergency department, 517 institutions that wished to participate took part in the surveillance, which represents an increase of 10 institutions from 507 institutions in 2024. The average number of reported cases per institution increased from 7.3 in 2024 to 8.6 in 2025. Cases subject to reporting involved patients who visited an emergency department and were diagnosed with a heat-related illness or died from a heat-related illness. Heat-related illnesses were classified into heat stroke (T67.0), heat cramps (T67.2), heat exhaustion (T67.3–T67.5), heat syncope (T67.1), heat edema (T67.7), and other heat-related illnesses (T67.8–T67.9) in accordance with the Korean Standard Classification of Diseases and Causes of Death.

Reporting is conducted in a manner wherein medical institutions submit daily reports and case reports through the “Disease and Health Integrated Management System.” Then, following approval by the competent public health center and city/province, the KDCA grants final approval to compile the data.

This report aims to analyze the surveillance data reported from May 15 to September 25, 2025, and present the key findings and implications.

## Results

Over the past 15 years (2011–2025), the annual average number of reported heat-related illness cases per 100 surveillance days reported through the “Heat-related Illness Emergency Department Surveillance System” was 1,634 (Figure 1) [6], and the annual average number of deaths was 17.8, with deaths occurring every year. In 2025, the total number of patients with heat-related illness reported through the “Heat-related Illness Emergency Department Surveillance System” was 4,460, representing a 20.4% increase compared with the previous year (3,704 cases in 2024), and the total number of estimated deaths from heat-related illness was 29, representing a 14.7% decrease compared with the previous year (34 deaths in 2024). Looking at the major meteorological conditions of the summer of 2025, the number of heatwave days was 29.7 days, a slight decrease compared with 2024 (30.1 days), but was the third-highest level since the operation of the surveillance system (Table 1) [6].

According to the Korea Meteorological Administration, the national average temperatures in June, July, and August were 22.9℃, 27.1℃, and 27.1℃, which were 1.5℃, 2.5℃, and 2.0℃ higher than the climatological normal temperature (1991–2020, 30-year average), respectively. During the summer (June–August), the national average temperature was 25.7℃, which was found to be 2.0℃ higher compared with the climatological normal temperature (1991–2020; 30-year average).

During the surveillance period, the highest number of patients with heat-related illness was recorded on July 8, 2025, with 259 patients in a single day. On this day, the national average daily maximum temperature was observed to be 34.1℃, and particularly in the Seoul area, it was a day when the temperature rose to as high as 37.8℃ (Figures 1, 2).

Regarding the major characteristics of patients with heat-related illness in 2025, the occurrence in males (3,553 patients, 79.7%) was approximately fourfold higher than in females (907 patients, 20.3%). By age group, most of the patients were in their 50s (865 patients, 19.4%), and 30.1% of the patients were elderly, aged 65 and over (1,341 patients). The number of patients per 100,000 population was calculated based on the Population Census data released by the Ministry of Data and Statistics in July 2025, and was the highest among those aged 80 and over at 19.9, showing an increasing trend as age increased.

By region, Gyeonggi-do had the highest number of cases with 978 patients (21.9%), followed by Gyeongbuk (9.8%), Gyeongnam (8.6%), and Seoul and Jeonnam (8.5%). The number of patients with heat-related illness per 100,000 population by region was the highest in Jeonnam at 21.4, followed by Ulsan and Gyeongbuk at 16.9 and Jeju at 15.8. By disease category, heat exhaustion accounted for 2,767 cases (62.0%), followed by heat stroke with 667 cases (15.0%), and heat cramps with 613 cases (13.7%).

Among all patients, the distribution of reported cases by location of occurrence showed that outdoors accounted for 3,534 patients (79.2%) and 23 estimated deaths (79.3%). Specifically, several cases occurred during outdoor work, including 1,431 individuals (32.1%) in workplaces and 542 individuals (12.2%) in paddy fields and dry fields (Table 2). The proportion by place of occurrence in Table 2 indicates the distribution of the locations of occurrence of reported patients with heat-related illness and does not represent a comparison of the fatality risk by location.

The total number of reported estimated deaths from heat-related illness was 29 (23 males and 6 females), and the cause of death for 93.1% (27 individuals) of them was estimated to be heat stroke. Among the estimated deaths, those aged 65 and over accounted for 58.6% (17 individuals), and the location of occurrence was outdoors for 23 individuals (79.3%) [7].

Comprehensive analysis of the occurrence patterns of heat-related illnesses reported in 2025 showed high proportions of cases (1,341, 30.1%) and deaths (17, 58.6%) among the elderly population aged 65 and older, and of outdoor cases (3,534, 79.2%) and deaths (23, 79.3%).

## Discussion

Heat-related illnesses caused by heatwaves can lead to severe harm, including death, if the response is delayed, but can be prevented by practicing basic health guidelines. During hot weather, it is advisable to consume fluids regularly before feeling thirsty and, when going out, to bring a hat or similar items to block sunlight and wear breathable clothing. In particular, during the hottest daytime hours (12:00–17:00), outdoor activities and workloads should be reduced and appropriate rest should be taken [7].

Older adults, infants and young children, individuals with chronic diseases, pregnant women, and those in environments where cooling is difficult are more vulnerable to heat, and thus require health management during heatwave periods. WHO states that outdoor and manual laborers are vulnerable to heat stress as they are exposed to excessive heat due to the nature of their work [3].

The 2025 analysis showed that those aged 65 and over accounted for 30.1% of all patients with heat-related illness and 58.6% of related deaths, with most patients with heat-related illness and deaths occurring outdoors, particularly in workplaces and paddy fields and dry fields. Outdoor workers are exposed to high-temperature environments for long periods, and due to the nature of their work, securing sufficient rest or a cooled environment can be difficult, necessitating continuous management to prevent heat-related illnesses. Accordingly, the KDCA promptly provides occurrence status and major characteristics through the surveillance system, and cooperation with relevant ministries and local governments is crucial.

In 2025, even though the number of heatwave days decreased slightly compared to the previous year, patients with heat-related illnesses increased by 20.4%, and a pattern of a short-term spike in patients on specific high-temperature days was observed. This finding suggests that when responding to heatwaves, rapid surveillance and response considering not only the number of heatwave days but also the intensity of high temperatures and patient occurrence trends are necessary. In addition, considering that occurrences were concentrated among the elderly and in outdoor workplaces, paddy fields, and dry fields, it is necessary to strengthen prevention activities targeting vulnerable groups, such as the elderly, outdoor laborers, and agricultural workers, as well as joint responses between relevant ministries and local governments. Data collected through the surveillance system can be utilized as evidence to identify vulnerable groups and risk periods early and to set priorities for prevention and response.

The government is establishing a cooperative system among relevant ministries, centered on the Ministry of the Interior and Safety (MOIS), to reduce casualties caused by heatwaves. MOIS established a pan-governmental heatwave response system in May 2026 to promote response tasks according to the functions of each ministry, and is strengthening responses to protect vulnerable groups and prevent health damage to the public [8].

However, this surveillance system aims to monitor trends in the occurrence of heat-related illnesses caused by heatwaves and rapidly share information to raise public awareness and encourage preventive activities. Since the system is based on reporting data from voluntarily participating medical institutions nationwide that operate an emergency department, it has the limitation that the collected data does not represent the overall status of heat-related illness occurrence.

## Figures and Tables

**Figure 1:**
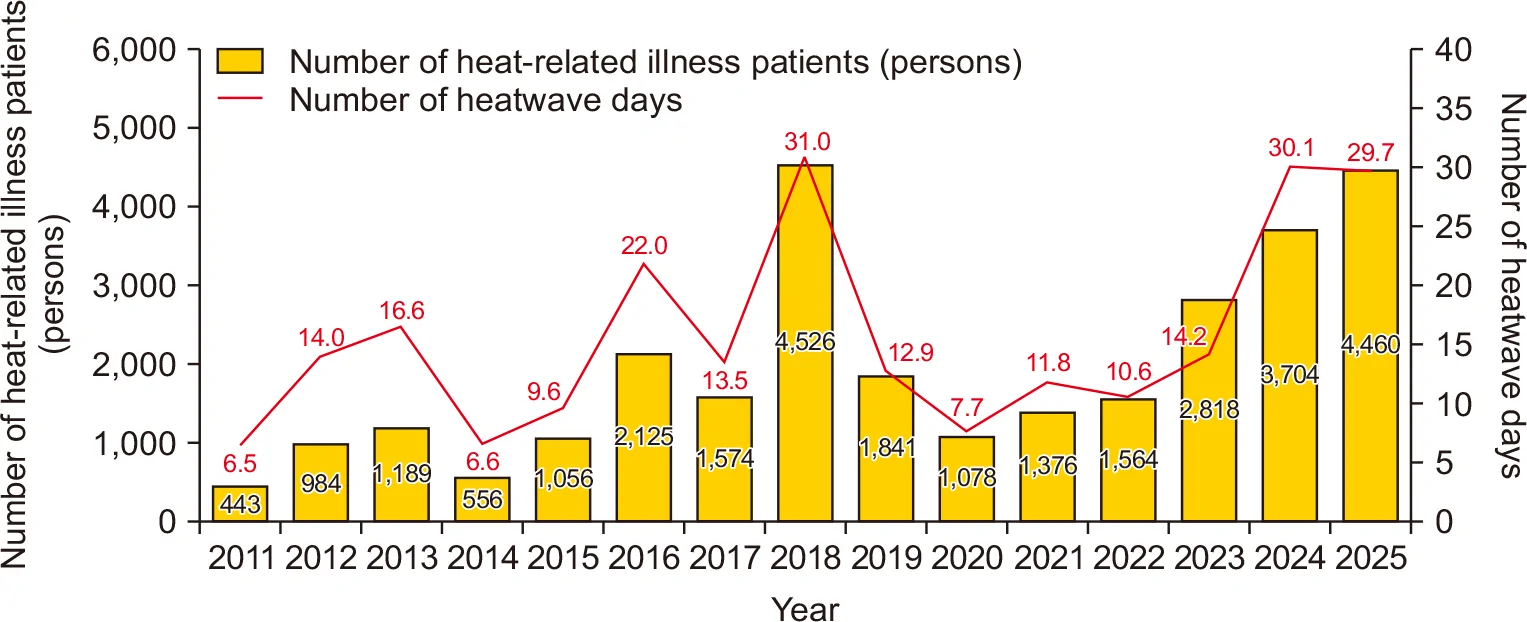
Annual number of heat-related illness patients and heatwave days Adapted from the article of Lee et al. (Public Health Wkly Rep 2024;17:1421-31) [6].

**Figure 2:**
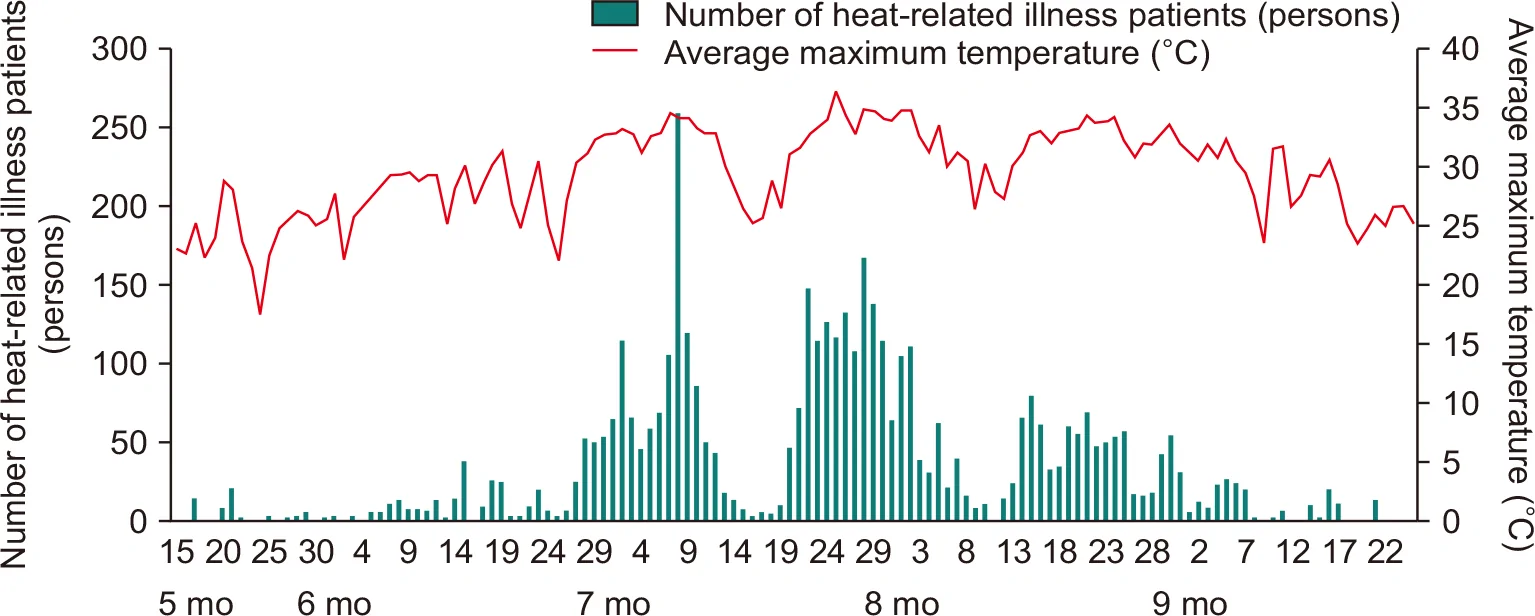
Daily heat-related illness patients and average maximum temperature in summer 2025

**Table 1:** Number of reported patients with heat-related illness, 2011–2025

Year	Monitoring period	Monitoring days	Patients with heat-related illnessa)	Estimated deaths	No. of heatwave daysb)
2011	July 1–September 3	65	443	6	6.5
2012	June 1–September 6	98	984	15	14.0
2013	June 2–September 7	98	1,189	14	16.6
2014	June 1–September 6	98	556	1	6.6
2015	May 24–September 5	105	1,056	11	9.6
2016	May 23–September 21	122	2,125	17	22.0
2017	May 29–September 8	103	1,574	11	13.5
2018	May 20–September 10	114	4,526	48	31.0
2019	May 20–September 20	124	1,841	11	12.9
2020	May 20–September 13	117	1,078	9	7.7
2021	May 20–September 30	134	1,376	20	11.8
2022	May 20–September 30	134	1,564	9	10.6
2023	May 20–September 30	134	2,818	32	14.2
2024	May 20–September 30	134	3,704	34	30.1
2025	May 15–September 25	134	4,460	29	29.7

Unit: persons or days. ^a)^Patients with heat-related illness include estimated deaths attributable to heat-related illness. ^b)^Source: Korea Meteorological Administration, Open MET Data Portal. Adapted from the article of Lee et al. (Public Health Wkly Rep 2024;17:1421-31) [6].

**Table 2:** Key results of the 2025 “Heat-related Illness Emergency Department Surveillance System”

Characteristic	Patients with heat- related illness (n=4,460)
Sex	
Male	3,553 (79.7)
Female	907 (20.3)
Age group (yr)	
0–9	9 (0.2)
10–19	132 (3.0)
20–29	445 (10.0)
30–39	608 (13.6)
40–49	603 (13.5)
50–59	865 (19.4)
60–69	834 (18.7)
70–79	485 (10.9)
≥80	479 (10.7)
Region	
Seoul	378 (8.5)
Busan	171 (3.8)
Daegu	140 (3.1)
Incheon	280 (6.3)
Gwangju	72 (1.6)
Daejeon	52 (1.2)
Ulsan	188 (4.2)
Sejong	33 (0.7)
Gyeonggi-do	978 (21.9)
Gangwon State	172 (3.9)
Chungcheongbuk-do	215 (4.8)
Chungcheongnam-do	227 (5.1)
Jeonbuk State	248 (5.6)
Jeollanam-do	381 (8.5)
Gyeongsangbuk-do	436 (9.8)
Gyeongsangnam-do	382 (8.6)
Jeju Special Self-Governing Province	107 (2.4)
Type of illness	
Heat stroke	667 (15.0)
Heat exhaustion	2,767 (62.0)
Heat cramps	613 (13.7)
Heat syncope	345 (7.7)
Heat edema	3 (0.1)
Other	65 (1.5)
Place of occurrence	
Indoor	926 (20.8)
Home	237 (5.3)
Building	137 (3.1)
Workplace	359 (8.0)
Greenhouse	53 (1.2)
Other indoor locations	140 (3.1)
Outdoor	3,534 (79.2)
Workplace	1,431 (32.1)
Playground (park)	237 (5.3)
Rice field/farm field	542 (12.2)
Mountain	119 (2.7)
Riverside/beach	52 (1.2)
Roadside	522 (11.7)
Around residence	186 (4.2)
Other outdoor locations	445 (10.0)

Unit: persons (%).
