# Supplementary material for: Results of Operating the 2025 “Heat-related Illness Emergency Department Surveillance System”
Source: Public Health Wkly Rep. 2026 Nov 5;19(42):1756–69. doi: 10.56786/PHWR.2026.19.42.2 (PMC13740384; doi:10.56786/PHWR.2026.19.42.2)
Supplement: Translation

# Translation: 2025년 「온열질환 응급실감시체계」 운영 결과

**Authors:** 지숙 채, 은지 정, 대식 안, 윤진 안

**Affiliations:** 질병관리청 건강위해대응관 기후보건ㆍ건강위해대비과

**Keywords:** 폭염, 온열질환, 열사병, 감시체계, 기후변화

## Abstract

**목적:**

기후변화로 인해 폭염의 빈도와 강도가 증가함에 따라 여름철 온열질환 발생 특성과 건강 영향을 분석하고 폭염으로 인한 국민 건강 피해를 최소화하기 위한 대응 및 예방 활동에 활용할 수 있는 분석자료를 도출하고자 한다.

**방법:**

질병관리청은 2011년부터 전국 응급실 운영 의료기관을 대상으로 「온열질환 응급실감시체계」를 운영하고 있다. 2025년에는 참여를 희망한 전국 517개(2025.5.15. 기준) 응급실 운영 의료기관을 대상으로 2025년 5월 15일부터 9월 25일까지 응급실에 내원한 온열질환자 및 추정 사망자 정보를 「질병보건통합관리시스템」을 통해 신고받았다. 수집된 자료를 바탕으로 성별, 연령별, 지역별, 질환별, 발생장소별 등 온열질환의 주요 발생 특성을 분석하여 매일 16시에 질병관리청 누리집을 통해 일일 현황을 제공하였다.

**결과:**

2025년 「온열질환 응급실감시체계」를 통해 신고된 온열질환자는 총 4,460명이었으며, 추정 사망자는 29명이었다. 이는 전년 대비 온열질환자는 20.4% 증가하고, 추정 사망자는 14.7% 감소한 수치였다. 신고된 환자는 남자 79.7% (3,553명)로 여자보다 많았고, 연령별로는 50대가 19.4% (865명)로 가장 많았다. 65세 이상 고령층은 전체 환자의 30.1% (1,341명)를 차지하였으며, 추정 사망자의 58.6% (17명)가 65세 이상에서 발생하였다. 질환별로는 열탈진이 62.0% (2,767명)로 가장 많았고, 발생장소는 실외가 79.2% (3,534명)로 실내보다 높게 나타났다. 특히 작업장(32.1%)과 논ㆍ밭(12.2%) 등 실외 작업 환경에서의 발생 비율이 높았다.

**결론:**

2025년 여름철 온열질환은 고령층과 실외 활동ㆍ작업 환경에서 집중적으로 발생한 것으로 나타났다. 기후변화로 폭염의 건강 영향이 확대될 것으로 예상됨에 따라 고령층 및 실외 작업자를 중심으로 한 예방 활동 강화와 관계 부처 협력을 통한 대응체계 마련이 필요하다. 또한 질병관리청은 「온열질환 응급실감시체계」 운영을 통해 수집된 정보를 신속히 제공함으로써 국민의 주의를 환기하고 예방활동을 유도할 필요가 있다.

**핵심요약**

**① 이전에 알려진 내용은?**

기후변화로 폭염의 빈도와 강도가 증가하면서 온열질환과 사망 위험이 커지고 있으며 질병관리청은 2011년부터 「온열질환 응급실감시체계」를 운영하고 있다.

**② 새로이 알게 된 내용은?**

2025년 여름철 온열질환자는 총 4,460명으로 전년 대비 20.4% 증가하였으며, 추정 사망자는 29명이었다. 고령층과 실외 작업 환경에서 발생 비율이 높게 나타났다.

**③ 시사점은?**

폭염 건강 피해는 고령층과 실외 작업자에게 집중되어 취약집단 대상 예방 강화와 신속한 정보 제공 및 대응체계 구축이 중요하다.

## 서 론

세계기상기구(World Meteorological Organization, WMO)에 따르면 2025년은 산업화 이전(1850–1900년) 대비 기온이 1.44℃±0.13℃ 상승하여 관측 이래 가장 더웠던 해 중 3위 안에 포함된다. 또한, 지난 11년(2015–2025년)은 모두 1850년 이후 관측 이래 가장 따뜻한 해로 기록되었으며, 특히 최근 3년(2023–2025년)은 역대 1–3위를 차지하며 이례적인 지구온난화 추세가 지속되고 있다[1,2].

2025년 우리나라는 6월 말부터 이른 더위가 시작되었고, 여름철(6–8월) 전국 평균기온은 25.7℃로 역대 가장 더웠던 2024년(25.6℃)보다 0.1℃ 높아 1973년 관측 이래 최고치를 경신하였다. 이는 평년 대비 2.0℃ 높은 수준이다[2].

2025년 세계보건기구(World Health Organization, WHO)와 WMO의 공동 보고서에 따르면 기후변화로 인해 폭염의 빈도와 강도가 증가함에 따라 위험한 고온 환경에 반복적으로 노출되는 근로자, 특히 농업ㆍ건설ㆍ어업 등 육체노동 종사자는 열사병, 탈수, 신장기능 장애, 신경계 질환 등의 건강 영향을 이미 경험하고 있는 것으로 나타났다. 또한 개발도상국의 취약계층(아동, 노인, 저소득층)에서도 심각한 건강 문제가 발생하고 있다고 보고하였다[3]. 폭염으로 인한 건강 피해는 개발도상국에 국한되지 않고 유럽과 일본 등에서도 광범위하게 보고되고 있다. 유럽 32개국에서는 2024년 여름 약 62,775명의 열 관련 사망이 발생한 것으로 추정되었으며[4] 일본에서는 2025년 5–9월 열중증으로 인한 응급이송자가 100,510명으로 2008년 조사 시작 이후 가장 많은 수준을 기록하였다[5].

질병관리청은 폭염으로 인한 국민 건강 피해를 최소화하기 위해 2011년부터 「온열질환 응급실감시체계」를 운영하고 있다. 또한, 온열질환자 발생 모니터링을 통해 신속한 발생 정보와 주요 특성을 질병관리청 누리집에 매일 공개함으로써 국민의 주의를 환기하고 예방수칙 실천을 유도하고 있다. 본 보고서는 2025년 여름철 「온열질환 응급실감시체계」의 주요 운영 결과와 시사점을 제시하고자 한다.

## 방 법

질병관리청은 2011년부터 「온열질환 응급실감시체계」를 운영하여 폭염으로 인한 온열질환 발생 추이와 취약집단 등 주요 특성을 신속히 파악하고 이를 지역별 및 주요 발생 특성(성별ㆍ연령별ㆍ직업별ㆍ질환별ㆍ발생 시간 및 장소)별로 정리하여 매일 16시에 누리집에 공개하고 있다.

2025년에는 전국 응급실 운영 의료기관 중 참여를 희망한 517개소가 감시에 참여하였으며 이는 2024년 507개소보다 10개소 증가한 규모이다. 기관 1개소당 평균 신고 건수는 2024년 7.3건에서 2025년 8.6건으로 증가하였다. 신고 대상은 응급실에 내원하여 온열질환으로 진단받거나 온열질환으로 사망한 경우로 하였으며, 온열질환은 한국표준질병ㆍ사인분류에 따라 열사병(T67.0), 열경련(T67.2), 열탈진(T67.3–T67.5), 열실신(T67.1), 열부종(T67.7) 및 기타 온열질환(T67.8–T67.9)으로 분류하였다.

신고는 「질병보건통합관리시스템」을 통해 의료기관이 일일보고와 사례보고를 실시하고 관할 보건소와 시ㆍ도의 승인 후 질병관리청이 최종 승인하여 자료를 집계하는 방식으로 이루어진다.

본 보고서는 2025년 5월 15일부터 9월 25일까지 신고된 감시자료를 분석하여 주요 결과와 시사점을 제시하고자 한다.

## 결 과

지난 15년간(2011–2025년) 「온열질환 응급실감시체계」로 신고된 연평균 감시일 100일당 온열질환자 신고건수는 1,634명이었고(그림 1) [6], 사망자는 17.8명으로 매년 지속적으로 발생하였다. 2025년 「온열질환 응급실감시체계」를 통해 신고된 온열질환자는 총 4,460명으로 전년 대비 20.4% 증가하였고(2024년 3,704명), 온열질환 추정 사망자는 총 29명으로 전년 대비 14.7% 감소하였다(2024년 34명). 2025년 여름철 주요 기상 상황을 살펴보면 폭염일수는 29.7일로 2024년(30.1일)에 비하여 다소 감소하였으나 감시체계 운영 이후 세 번째로 높은 수준이었다(표 1) [6].

기상청에 따르면 6월 전국 평균기온은 22.9℃로 평년(1991–2020년, 30년 평균) 대비 1.5℃ 높았고, 7월은 27.1℃로 2.5℃ 높았으며, 8월은 27.1℃로 2.0℃ 높았다. 여름철(6–8월) 전국 평균기온은 25.7℃로 평년(1991–2020년, 30년 평균) 대비 2.0℃ 높은 것으로 나타났다.

감시기간 중 온열질환자가 가장 많이 발생한 날은 2025년 7월 8일로 하루 259명이 발생하였다. 이날 전국 평균최고기온이 34.1℃로 관측되었고 특히 서울 지역은 37.8°C까지 기온이 올랐던 날이었다(그림 1, 2).

2025년 온열질환자 주요 특성으로는 남자(3,553명, 79.7%)가 여자(907명, 20.3%)보다 약 4배 많이 발생하였다. 연령별로는 50대가 865명(19.4%)으로 가장 많았고, 65세 이상 고령층이 전체 환자의 30.1% (1,341명)를 차지하였다. 인구 10만 명당 환자 수는 국가데이터처가 2025년 7월 발표한 인구총조사 자료를 기준으로 산출하였으며 80세 이상에서 19.9명으로 가장 높아 연령이 증가할수록 높아지는 경향을 보였다.

지역별로는 경기도가 978명(21.9%)으로 가장 많았고 경북(9.8%), 경남(8.6%), 서울ㆍ전남(8.5%) 순으로 나타났다. 인구 10만 명당 지역별 온열질환자 수는 전남 21.4명, 울산ㆍ경북 16.9명, 제주 15.8명 순으로 나타났다. 질환별로는 열탈진이 2,767명(62.0%), 열사병은 667명(15.0%), 열경련 613명(13.7%) 순으로 나타났다.

전체 환자 중 발생장소별 신고 분포에서는 실외가 환자 3,534명(79.2%), 추정 사망자 23명(79.3%)으로 나타났다. 세부적으로는 작업장 1,431명(32.1%), 논ㆍ밭 542명(12.2%) 등 실외 작업 중 발생한 사례가 많았다(표 2). 표 2의 발생장소별 비율은 신고된 온열질환자의 발생장소 분포를 나타낸 것으로, 장소별 치명 위험을 비교한 결과는 아니다.

신고된 온열질환 추정 사망자는 총 29명(남성 23명, 여성 6명)으로 이들 중 93.1% (27명)의 사망원인이 열사병으로 추정되었다. 추정 사망자 중 65세 이상이 58.6% (17명)이었고, 발생장소는 실외가 23명(79.3%)이었다[7].

2025년 신고된 온열질환 발생 양상을 종합해 보면, 65세 이상 고령층의 환자 발생(1,341명, 30.1%)과 사망(17명, 58.6%), 그리고 실외에서의 환자 발생(3,534명, 79.2%)과 사망(23명, 79.3%)의 비율이 높게 나타났다.

## 논 의

폭염으로 발생하는 온열질환은 대응이 지연될 경우 사망 등 중대한 피해로 이어질 수 있으나, 기본적인 건강수칙을 실천하면 예방이 가능하다. 더운 날씨에는 갈증을 느끼기 전에 수분을 규칙적으로 섭취하고 외출 시에는 햇빛을 가릴 수 있는 모자 등을 챙기고 통기성이 좋은 옷을 착용하는 것이 바람직하다. 특히, 기온이 가장 높은 낮 시간대(12–17시)에는 야외활동과 작업량을 줄이고 적절한 휴식을 취해야 한다[7].

고령자와 영유아, 만성질환자, 임신부, 냉방이 어려운 환경에 있는 사람은 열에 더욱 취약하므로 폭염 기간 동안 건강관리가 필요하다. WHO는 실외 및 육체노동 종사자는 업무 특성상 과도한 열에 노출되어 열 스트레스에 취약하다고 명시하고 있다[3].

2025년 분석 결과에서 65세 이상 고령자는 전체 온열질환자의 30.1%, 사망자의 58.6%를 차지하였으며, 대부분의 온열질환자 및 사망자가 실외에서 발생한 것으로 나타났으며 특히 작업장과 논ㆍ밭에서의 발생이 많았다. 실외 작업자는 고온 환경에 장시간 노출되고 작업 특성상 충분한 휴식이나 냉방 환경 확보가 어려울 수 있어 온열질환 예방을 위한 지속적인 관리가 필요하다. 이에 질병관리청은 감시체계를 통해 발생 현황과 주요 특성을 신속히 제공하고 관계 부처 및 지자체와의 협력이 중요하다.

2025년에는 폭염일수가 전년보다 다소 감소하였음에도 온열질환자는 20.4% 증가하였으며 특정 고온일에 환자가 단기간 급증하는 양상이 나타났다. 이는 폭염 대응 시 폭염일수뿐만 아니라 고온의 강도와 환자 발생 추이를 함께 고려한 신속한 감시와 대응이 필요함을 시사한다. 또한 65세 이상 고령층과 실외 작업장ㆍ논ㆍ밭에서 발생이 집중된 점을 고려할 때, 고령자와 실외 근로자ㆍ농업인 등 취약집단을 대상으로 한 예방활동과 관계부처 및 지자체 간 연계 대응을 강화할 필요가 있다. 감시체계를 통해 수집되는 자료는 취약집단과 위험 시기를 조기에 파악하고 예방ㆍ대응의 우선순위를 설정하는 근거로 활용될 수 있다.

정부는 폭염으로 인한 인명피해를 줄이기 위해 행정안전부를 중심으로 관계 부처 간 협력체계를 마련하고 있다. 행정안전부는 2026년 5월 범정부 폭염 대응체계를 마련하여 부처별 기능에 따른 대응 과제를 추진하고 취약계층 보호 및 국민의 건강 피해 예방을 위한 대응을 강화하고 있다[8].

다만, 본 감시체계는 폭염으로 인한 온열질환 발생 동향을 모니터링하고 신속하게 정보를 공유하여 국민의 주의 환기 및 예방 활동 유도에 목적이 있으며 전국 응급실 운영 의료기관 중 자발적으로 참여한 기관의 신고자료를 기반으로 하므로 수집된 자료가 전체 온열질환 발생 현황을 의미하지 않는 제한점이 있다.

## Figures and Tables

**그림 1:** 연도별 온열질환자 수와 폭염일수 Adapted from the article of Lee et al. (Public Health Wkly Rep 2024;17:1421-31) [6].

**그림 2:** 2025년 여름철 일별 온열질환 발생과 평균 최고기온

**표 1:** 연도별 감시체계 운영 결과(2011–2025년)

연도	운영 기간	감시일수	온열질환자a)	추정 사망자	폭염일수b)
2011	7.1.–9.3.	65	443	6	6.5
2012	6.1.–9.6.	98	984	15	14.0
2013	6.2.–9.7.	98	1,189	14	16.6
2014	6.1.–9.6.	98	556	1	6.6
2015	5.24.–9.5.	105	1,056	11	9.6
2016	5.23.–9.21.	122	2,125	17	22.0
2017	5.29.–9.8.	103	1,574	11	13.5
2018	5.20.–9.10.	114	4,526	48	31.0
2019	5.20.–9.20.	124	1,841	11	12.9
2020	5.20.–9.13.	117	1,078	9	7.7
2021	5.20.–9.30.	134	1,376	20	11.8
2022	5.20.–9.30.	134	1,564	9	10.6
2023	5.20.–9.30.	134	2,818	32	14.2
2024	5.20.–9.30.	134	3,704	34	30.1
2025	5.15.–9.25.	134	4,460	29	29.7

단위: 명 또는 일. ^a)^온열질환자는 ‘온열질환 추정 사망자’를 포함하는 수치임. ^b)^기상청 기상자료개방포털. Adapted from the article of Lee et al. (Public Health Wkly Rep 2024;17:1421-31) [6].

**표 2:** 2025년 「온열질환 응급실감시체계」 주요 결과

특성	온열질환자(n=4,460)
성별	
남성	3,553 (79.7)
여성	907 (20.3)
연령별(세)	
0–9	9 (0.2)
10–19	132 (3.0)
20–29	445 (10.0)
30–39	608 (13.6)
40–49	603 (13.5)
50–59	865 (19.4)
60–69	834 (18.7)
70–79	485 (10.9)
≥80	479 (10.7)
지역별	
서울특별시	378 (8.5)
부산광역시	171 (3.8)
대구광역시	140 (3.1)
인천광역시	280 (6.3)
광주광역시	72 (1.6)
대전광역시	52 (1.2)
울산광역시	188 (4.2)
세종특별자치시	33 (0.7)
경기도	978 (21.9)
강원특별자치도	172 (3.9)
충청북도	215 (4.8)
충청남도	227 (5.1)
전북특별자치도	248 (5.6)
전라남도	381 (8.5)
경상북도	436 (9.8)
경상남도	382 (8.6)
제주특별자치도	107 (2.4)
질환별	
열사병	667 (15.0)
열탈진	2,767 (62.0)
열경련	613 (13.7)
열실신	345 (7.7)
열부종	3 (0.1)
기타	65 (1.5)
발생장소	
실내	926 (20.8)
집	237 (5.3)
건물	137 (3.1)
작업장	359 (8.0)
비닐하우스	53 (1.2)
기타	140 (3.1)
실외	3,534 (79.2)
작업장	1,431 (32.1)
운동장(공원)	237 (5.3)
논ㆍ밭	542 (12.2)
산	119 (2.7)
강가, 해변	52 (1.2)
길가	522 (11.7)
주거지 주변	186 (4.2)
기타	445 (10.0)

단위: 명(%).
